# Artificial Intelligence-Driven Neural Computing for Navigation Decision Classification in Autonomous Vehicles

**DOI:** 10.3390/biomimetics11100705

**Published:** 2026-10-03

**Authors:** Mohammed Baihan

**Affiliations:** Department of Computer Science, College of Computer and Information Sciences, King Saud University, Riyadh P.O. Box 11451, Saudi Arabia; malbaihan@ksu.edu.sa

**Keywords:** autonomous vehicles, navigation, sensor-kinematics data, deep learning, optimization

## Abstract

Autonomous vehicles (AVs) require robust decision-making capabilities to navigate dynamic environments securely and effectively. Furthermore, advanced sensors and computational models are utilized to perceive the environment and predict the behavior of other road users. The main challenges include dealing with unpredictable factors such as weather fluctuations, traffic patterns, and road obstacles. While these technologies provide contextual awareness, existing techniques often face difficulties in handling complex sensor data under rapidly changing conditions and selecting the most useful features for navigation decision classification. To overcome these challenges, there is a need for reliable and intelligent architectures for navigation decision classification systems to support AVs by utilizing AI-driven machine learning (ML) and deep learning (DL) techniques. Currently, DL methods have gained significant importance in enhancing AV capabilities by enabling improved perception and decision classification from complex data. Therefore, this paper introduces an Intelligent Bidirectional Temporal Attention Framework for Navigation Decision Classification in Autonomous Vehicles (IBTAF-NDCAV). The major aim of this research is to design an effective navigation decision classification system for classifying driving actions using sensor data. To accomplish this aim, the proposed method applies improved mutual information-based feature selection to select informative features while reducing redundant ones. For classification, the proposed IBTAF-NDCAV method utilizes a bidirectional temporal attention convolutional network as an AI-driven neural computing model that captures temporal dependencies and effectively classifies navigation decisions. To enhance convergence and training stability, the Adamax optimizer is used to optimize the neural network training process. The experimental result analysis of the IBTAF-NDCAV technique is conducted on a benchmark Autonomous Driving Sensor-Kinematics dataset, which includes 5000 samples distributed across five classes: follow**,** yield**,** stop**,** lane change, and overtake. The comparative results demonstrate superior performance, achieving an accuracy of 96.76%, with class-wise precision, recall, F1-score values and MCC of 90.97%, 81.68, 85.31%, and 83.40% on the 20% testing set. The temporal attention mechanism highlights informative temporal patterns contributing to the navigation decisions, thus providing a basis for interpreting the model’s decision-making procedure. Therefore, the result highlights the effectiveness of the model, making it appropriate for utilization in autonomous vehicle navigation and path-planning applications.

## 1. Introduction

Autonomous driving (AD) is a significant component of smart transportation, offering the capacity to improve road safety, mobility, and transportation efficacy. Behavioral decision-making plays a vital role in the higher-level smart driving model of intelligent vehicles and is a significant focus of recent smart transportation system studies [1]. The main challenge in AD lies in managing unexpected circumstances that are not represented in the training data, termed out-of-label (OOL) dangers, which include atypical road situations, rare objects, visually degraded conditions, and low-light environments [2]. These dangers often occur at longer distances, during partial occlusion, under poor illumination, and in uncommon semantic contexts and are inadequately represented in standard closed-set databases. Therefore, perception methods fail to accurately detect complex safety risks [3]. Furthermore, AD systems encounter significant limitations in real-time scenarios, especially regarding safe navigation in adverse weather and interactions with vulnerable road users. Severe weather conditions, including snow, glare, rain, mist, fog, and haze, could significantly affect the navigation and perception performance of sensor-based systems [4]. These conditions are considered practical challenges for autonomous navigation; however, they are not separately evaluated in this study, which focuses on sensor-kinematic navigation decision classification using the selected benchmark dataset.

The autonomous navigation model of smart surface vessels and self-driving cars constitutes significant achievements, serving as low-cost unmanned transportation. With advanced perception and decision-making capabilities, autonomous operations increase operational efficiency, enable safe navigation, and promote reduced labor dependency [5]. Autonomous navigation capabilities serve as the technological foundation of self-driving vehicles and smart surface vessels. On the other hand, efficient self-driving vehicle technology requires high computational performance for real-time autonomous navigation, resulting in limited development progress in self-driving vehicles and smart surface vehicles [6]. Moreover, owing to limited human experience and significant data acquisition needs, current navigation models face difficulties in attaining dependable results under complex and dynamically varying driving conditions [7]. The low efficiency of the navigation method, the lack of flexibility in handling intricate navigation conditions and unknown scenarios, and the unpredictability of controller switching are not efficiently resolved, which restricts the practical implementation of AD technology [8].

The motion planning and decision-making algorithms for self-driving vehicles combine theories from numerous disciplines with machine learning (ML), intelligent optimization, nonlinear control, and pattern recognition [9]. ML models, especially deep learning (DL) methods, efficiently enhance the capability of encoding complex model characteristics. These systems offer the basis for safe interactions between self-driving vehicles and other road users [10]. Moreover, planning and decision-making algorithms should examine legal and ethical responsibilities, maintain moral standards, and comply with traffic laws in emergency conditions [11]. Recent studies on planning and decision-making methods concentrate on enhancing robustness, improving stability and safety in unexpected conditions, and improving the predictive accuracy of the external environment and other traffic participants. Moreover, researchers are working to minimize computational resource consumption and enhance algorithmic efficacy for rapid responses in resource-limited scenarios [12]. In this study, the problem focuses on sensor-based driving-action classification for autonomous vehicle navigation, considering five actions: Follow, Yield, Stop, Lane Change, and Overtake. For practical vehicle deployment, navigation decision classification models should also consider computational efficiency and response time while maintaining reliable classification performance.

### Motivation and Research Contributions 

Autonomous vehicles are capable of making complex, dynamic decisions in unpredictable environments, whereas current navigation decision classification methods typically process basic measurements of distance, position, and speed, failing to capture finer contextual nuances such as rare hazards, adverse weather conditions, and traffic congestion. Traditional datasets cannot fully capture the dynamic dependencies between kinematic sensors and the dynamic temporal correlations among sensors, resulting in a loss of the model’s understanding of the current driving status. Bidirectional temporal modeling is adopted to learn contextual data from both subsequent and preceding observations within the sensor sequence, thus maintaining the distinction among independent driving actions. This imposes limitations on the overall effectiveness and robustness of existing models in establishing safer and optimal paths to fulfill driving goals. Consequently, in view of these shortcomings, this study proposes an Intelligent Bidirectional Temporal Attention Framework for Navigation Decision Classification in Autonomous Vehicles (IBTAF-NDCAV) system. The presented IBTAF-NDCAV system incorporates feature processing, data preprocessing and selection, and the expansion of a bidirectional attentive deep learning classifier model with refined parameters. The performance of the proposed IBTAF-NDCAV model is evaluated on a standard dataset through distinct evaluations using several different parameters, and this study’s major contributions are enumerated as follows.

▪ It integrates an improved mutual information (MI)-based feature selection (FS) method to identify the most informative features and capture significant patterns from complex data.
▪ It designs a bidirectional temporal attention convolutional network (Bi-TACN) to learn temporal features from sensor data, enabling navigation decision actions such as follow, yield, stop, lane change, and overtake.
▪ It utilizes the Adamax optimizer to improve convergence stability and training performance of the proposed neural network with limited computational complexity.
▪ It conducts comprehensive evaluations on the benchmark Autonomous Driving Sensor-Kinematics dataset, and the comparative study indicates the improved performance of the proposed IBTAF-NDCAV methodology across diverse performance measures.

## 2. Literature Review

This section reviews previous research works on navigation decision classification systems in autonomous vehicles. Almadhor et al. [13] introduced a DL-driven structure that combines multimodal sensor fusion, digital twin simulation, sophisticated 3D object detection, and XAI to improve the self-driving vehicle perception and reasoning. A ResNet-50 deep convolutional backbone is used to extract high-level spatial features, while a transformer-driven model integrates temporal context. Huang et al. [14] proposed an end-wise DNN that takes visual images and corresponding depth data as input at an earlier fusion level and outputs pixel-wise semantic segmented data. Sethuraman et al. [15] presented the improved ensemble weighted support vector-driven adaptive crossover black widow method for cloud computing (CC) in self-driving vehicles. In addition, the framework uses an ensemble model to improve classification performance and the AdaBoost method to enable object detection. 

Gondhalekar et al. [16] introduced a novel AD framework extending from highways to urban areas by integrating DL with RL and hybrid AI techniques. The approach performs object detection, accurate perception, and collision avoidance in difficult traffic scenarios using sensor fusion with advanced computer vision, LiDAR, and radar. In addition, it involves resource-efficient AI techniques personalized for lower-power hardware, increasing the sustainability of autonomous technology. Mohata et al. [17] presented an innovative, strong, and smart navigation structure for vehicles through the combined fusion of sophisticated models, such as analytical prediction and remote sensing, for precise obstacle and object detection. ViewVerse, RuleRise, and TaskTrek create an integrated approach for understanding vehicle-environment interactions, and the research not only helps to accelerate the growth of accurate navigation-powered autonomous vehicles but also supports friendly mobility and motion-tracking solutions. Sugumar et al. [18] presented an IoT-based Tabu Search-driven Chaotic Dragonfly Optimizer method integrated with a multi-hypergraph-based compressed GRU and a BiRNN-based dual deep Q-Learning technique, intending to assure the comfort and safety of self-driving vehicles in real-world driving situations. The proposed model integrates an asymmetric convolutional layer to minimize the model parameters and computational requirements while preserving spatial data. Aloqaily et al. [19] explored a method for an intrusion detection model for protecting internal vehicle interactions from possible threats and ensuring safe data transmission. In this method, a real automotive network dataset with DoS, Fuzzy, and Spoofing attacks was utilized. Ge et al. [20] introduced an algorithm called Deep Reinforcement Learning Navigation via Decision Transformer (DRLNDT) to improve the decision-making capabilities of autonomous vehicles operating in partially observable urban environments. Hu et al. [21] presented Unified Autonomous Driving (UniAD), a comprehensive framework that integrates full-stack autonomous driving tasks into a single network. It is designed to use the advantages of each module and provide complementary feature representations for agent interaction from a global perspective. Table 1 summarizes recent approaches reported in the literature, emphasizing the datasets used, model architectures, and key findings relevant to autonomous driving.

Although existing studies show the potential of deep learning, transformer-based models and hybrid optimization techniques for autonomous driving, some research gaps still remain. Transformer-based methods that use temporal information have mainly been applied to perception and scene understanding, while sensor-based navigation decision classification has received comparatively less attention. These gaps motivate the development of a task-specific framework that combines informative sensor feature selection, temporal dependency modeling, and computationally efficient optimization for navigation decision classification. Furthermore, many methods do not adequately address the feature selection (FS) of the most relevant sensor features or learn the bidirectional temporal dependencies in time-series sensor data. The combination of these components is intended to provide complementary benefits by reducing redundant sensor information, capturing temporal context, and supporting stable model training. Its effectiveness is evaluated through experimental comparison.

## 3. Proposed Framework

**Figure 1:**
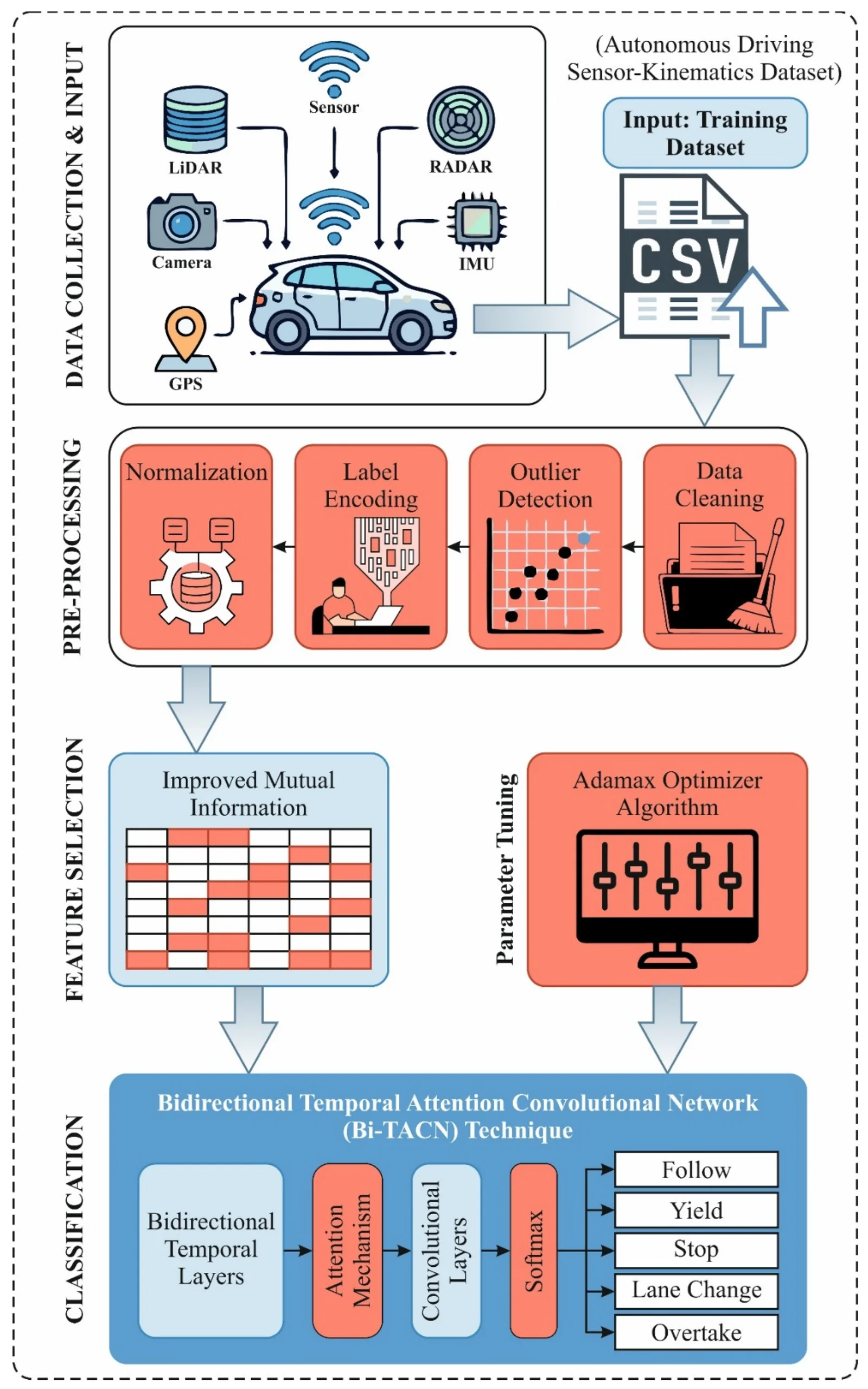
Overall structural design of the IBTAF-NDCAV module.

This study follows a systematic approach for classifying decision actions using sensor data that combines the process of data preprocessing, FS, bidirectional attention-based classification, and parameter tuning. Figure 1 depicts the overall structural design of the IBTAF-NDCAV framework. In the first stage, data preprocessing is performed using data cleaning, outlier detection, label encoding, and Z-score normalization to improve data quality for analysis. Next, the improved MI-based FS is applied to select the most discriminative features, thereby eliminating redundant ones. Following feature selection, a Bi-TACN model is employed for effective navigation decision classification. Parameter tuning is then carried out using the Adamax optimizer to improve training stability. Finally, the proposed model is evaluated using a benchmark Autonomous Driving Sensor-Kinematics dataset to demonstrate its superior performance.

### 3.1. Data Preprocessing Module

Data cleaning, outlier detection, label encoding, and standardization are performed to ensure data quality and make the collected data more suitable for model training.

**Data Cleaning:** Raw sensor and kinematic data may contain noise and unusual values that can affect the performance of the classifier [22]. Outliers can be identified using the Z-score and IQR methods and removed from the dataset. Categorical data are converted into numerical values for classification, while sensor data with different scales are standardized using Z-score normalization. Both Z-score and IQR criteria were considered to identify statistically unusual observations during preprocessing. The selected outlier-detection procedure was applied consistently to the dataset. However, the two methods were not separately compared, as outlier detection was treated as part of the preprocessing stage rather than as a component of the proposed model.

In practical autonomous vehicles, sensor data may contain missing, noisy, or inconsistent values due to measurement errors, communication interruptions, or changing environmental conditions.

**Outlier Detection:** Kinematic and sensor data may contain unusually low or high values that can affect classifier performance. The Z-score and IQR methods can be used to detect and remove such outliers, leaving typical data that can improve the stability and reliability of classification. To avoid removing potentially meaningful driving events, outlier removal was performed only on statistically anomalous sensor and kinematic observations. Complete driving-action classes or rare navigation events were not removed during preprocessing. Samples from all five driving-action classes—Follow, Yield, Stop, Lane Change, and Overtake—were retained in the dataset.

**Label Encoding:** Categorical features describing driving actions or navigational choices are encoded as numerical values to make them suitable for the classification model. Every category *C_k_* among the *K* classes receives a separate integer value. This encoding enables the model to process categorical data during the training process. By encoding labels as numerical values, the model can understand class information accurately. This encoding method maintains the unique identity of all classes without creating any bias in the model. The encoded class labels *y*_encoded_ are utilized as targets for model learning and performance evaluation. The label encoding process is given below.

*y*_encoded_ = *f*(*C_k_*) ∈ {0,1,2,…,*K* − 1} (1)

**Z-Score Normalization:** Standardizing with Z-scores indicates the position of an observation in the data relative to the mean, measured in terms of standard deviations. Additionally, it is especially appropriate for techniques that require standardized data.

(2) $$Z=\frac{x-\mu }{\sigma }\;$$

In this expression, *μ* implies the mean, *σ* denotes the standard deviation (SD), and *χ* is a specific data value.

No samples were excluded according to their driving-action class. Instead, the outlier analysis was performed only on sensor and kinematic features to identify statistically unusual observations, while retaining samples from all five navigation decision classes. The thresholds used for outlier detection were defined based on the feature distributions in the selected dataset. Therefore, these thresholds may not be directly suitable for other datasets or driving conditions and would need to be adjusted according to their sensor characteristics and data distributions.

### 3.2. Improved Mutual Information-Based Feature Selection

An enhanced MI-based FS method is employed to identify highly relevant features by evaluating their dependency on navigation decisions [23]. Redundant and less significant features are eliminated, reducing noise and enhancing data quality, which ensures that the model focuses on critical features and improves classification. In the present study, the improved MI-based feature selection method selects 16 informative features from the original 18 features, while the remaining two redundant or less informative features are eliminated for subsequent classification. In the case of two discrete variables, MI quantifies the amount of shared information between them. The formula for MI is provided in the following equation.

(3) MIU,V=HU−HUV=∑u∈U∑v∈Vpu,vlog p(u,v)pupv

In this formulation, the entropy of *U* is depicted as *H*(*U*), and *H*(*U*| *V*) denotes the conditional entropy of *U* given *V*. The marginal probabilities of *A* and *B* are given by *p*(*u*) and *p*(*v*), whereas *p*(*u*,*v*) defines their joint probability distribution. Equation (3) is applied to compute the entropy *H*(*U*).

(4) HU=−∑ui∈Upuilog puj

Equation (5) provides the formulation for calculating the conditional entropy *H*(*U*∣*V*).

(5) HUV=−Σvj∈VpvjΣui∈Upuivjlog puivj

Equation (6) denotes the feature selection objective function that combines feature relevance, redundancy, and conditional redundancy.

(6) $$J(U_{k})=I(U_{k;}\cdot V)-\beta \sum_{U_{j}\in S}I(U_{j;}\cdot U_{k})+\gamma \sum_{U_{j}\in S}I(U_{j;}\cdot U_{k}|V)$$

Unlike conventional MI-based feature selection, the proposed improved MI-based FS incorporates the conditional redundancy term *I*(*U_j_*_;_ ⋅ *U_k_*|*V*), which considers the dependency between candidate features in relation to the class label *V*. This additional term helps to reduce class-dependent redundancy among the selected features while preserving their relevance to the navigation decision classes, thereby improving the selection of informative sensor-kinematics features for navigation decision classification.

This formulation consists of both a relevancy term and redundancy-related terms, which are detailed in Equations (7) and (8), respectively. Equation (7) defines the relevancy term, and the redundancy component is calculated as the addition of marginal redundancy and conditional redundancy, each scaled by the parameters *β* and *γ*, where 0 ≤ *β*, *γ* ≤ 1.

*I*(*U_k_*_;_*V*) (7)

(8) $$\beta \sum_{U_{j}\in s}I(U_{j},U_{k})+\gamma \sum_{U_{j}\in s}I(U_{j},U_{k}|V)$$

(9) $$\beta \sum_{U\in s}I\left(U_{j;}U_{k}\right)$$

(10) $$\sum_{U\in s}I\left(U_{j;}U_{k}|V\right)$$

The conditional MI between *U_k_* and another feature, *U_j_*, given the selected label *V* is represented by *I*(*U_j_*; *U_k_*∣*V*), and the measure *I*(*U_k_*; *V*) indicates the MI between the candidate feature *U_k_* and the class *V*.

### 3.3. Bidirectional Attention Convolutional-Based Classification

The Bi-TACN model incorporates dilated causal convolution, residual connections, a bidirectional structure, and channel attention to effectively capture temporal features from sensor-kinematics data [24]. The Bi-TACN identifies temporal dynamics in sensor and kinematic data by utilizing dilated causal convolutions and residual structures. The model employs an Efficient Channel Attention (ECA) module and a bidirectional design to better identify and represent important driving features, improving navigation decision classification. The Bi-TACN architecture consists of three TCN layers, with each layer using 64 filters, a kernel size of 3, and a dilation rate of 2. ReLU activation, same padding, and a stride of 1 are applied throughout the TCN layers. Residual connections are used to improve information flow, while pooling is not applied to maintain the temporal resolution of the sensor sequences. The attention module uses four heads to capture different temporal dependencies from the learned representations. These parameter settings were chosen through preliminary experiments to provide a suitable balance between temporal representation and computational complexity. Below is a detailed description of the primary components and functionalities of the Bi-TACN model.

Dilated Causal Convolution: The standard convolutional model faces difficulties when handling time series data. In particular, while capturing local features at a specific moment, the output at that time may be affected by data from succeeding time steps. Such behavior breaks the natural temporal ordering of features in the time series. The module is used to preserve the temporal order of the input sequence while capturing a wider temporal context. The module consists of an input layer followed by convolutional processing and an output layer. Given a sequence of data *X* = {*x*_1_, *x*_2_, …, *x_T_*}, feature extraction at time step *t* is restricted to values from {*x*_1_, *x*_2_, …, *x_t_*} and is not influenced by future elements {*x_t_*_+1_, *x_t_*_+2_, …, *x_T_*}. This is ensured by causal convolution, which uses unilateral padding or one-sided padding to keep the input and output similar in sequence length, preventing the model from accessing upcoming data during prediction, which is represented as

(11) $$\left(F\times X\right)\left(x_{t}\right)=\sum_{k=1}^{K}f_{k^{\chi }t-K+k}$$

Here, *x_t_*_−*K*+*k*_ denotes the sampled component, *F* = {*f*_1_, *f*_2_, …, *f_K_*} indicates the causal convolution filter, and *K* represents the filter size. Nevertheless, the receptive field in causal convolution grows only when the convolution kernel size is increased. As larger kernel sizes lead to higher parameter complexity, dilated convolution is used within causal convolution to address this issue. The dilated convolution kernel captures feature data at fixed intervals (or spacing), thereby expanding the range of data it can extract. In addition, the dilated kernel maintains the original size of the causal convolution kernel.

(12) $$\left(F_{d}\times X\right)\left(x_{t}\right)=\sum_{k=1}^{K}f_{k^{\chi }t-\left(K-k\right)d}$$

**Figure 2:**
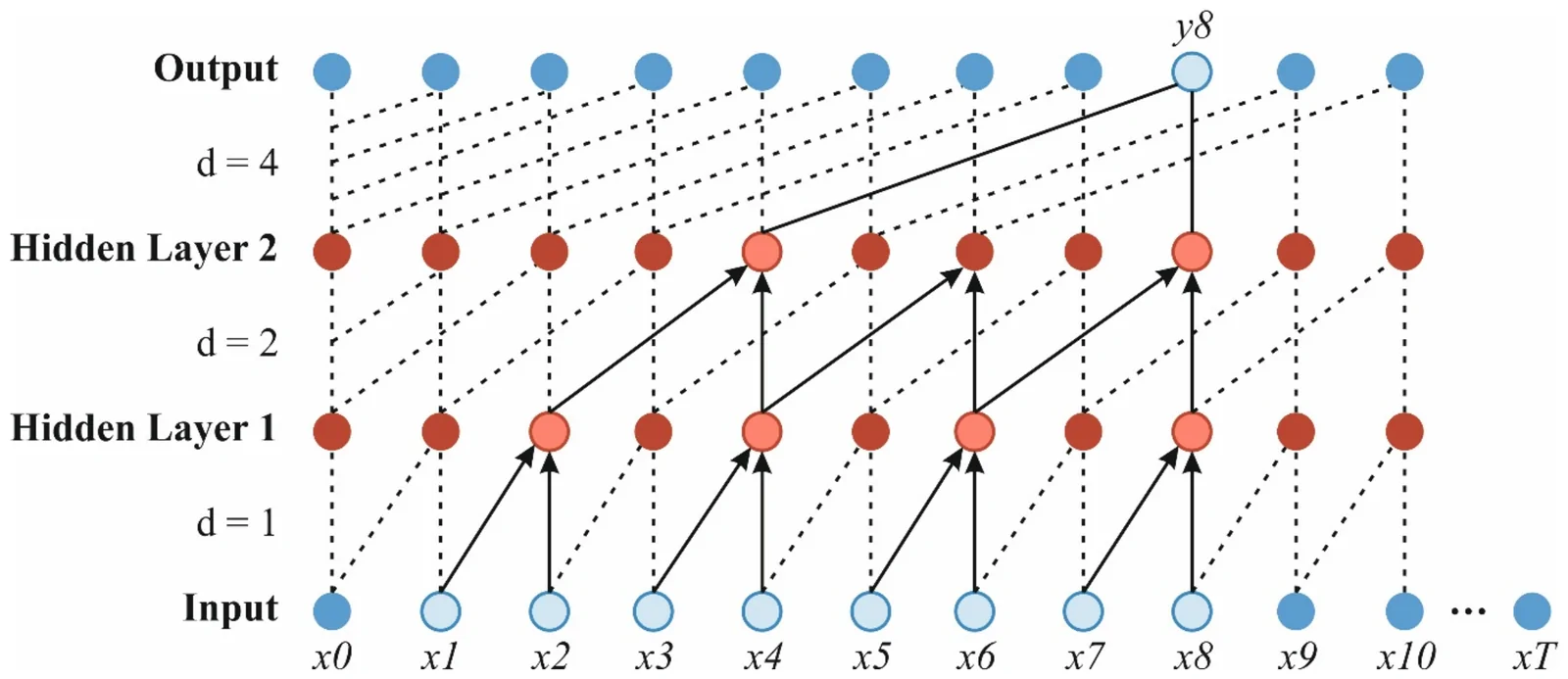
Structure of the dilated causal convolution module.

Here, *x_t_*_−(*K*−*k*)*d*_ is the element after interval sampling, *d* is the dilation rate, and *F_d_* = {*f*_1_, *f*_2_, …, *f_K_*} denotes the dilated convolution filter. Figure 2 displays the structure of the dilated causal convolution module.

Residual Structure: Increasing network depth makes the training process more challenging. The primary cause of this issue is the vanishing gradient phenomenon, often encountered in deep networks during multilayer backpropagation of error signals. This framework provides an effective solution to the issues faced during deep network training.

*x_l_*_+1_ = *x_l_* + *H*(*x_l_*) (13)

In this formulation, *H*(*x_l_*) indicates the residual mapping, and *x_l_* implies the output of the *l*-th layer, which incorporates dilated causal convolutions combined with activation functions. As described in Equation (13), the input bypasses feature representation operations and can be directly combined with the output. In addition, the residual mapping helps accelerate model convergence while enhancing stability and supporting more effective optimization.

Bidirectional Structure: The model mentioned above uses only one-directional sensor information, which results in partial feature representation. To overcome this shortcoming, a bidirectional structure is employed. It evaluates each time step from both forward and backward temporal perspectives, allowing it to extract global characteristics of the time series. The bidirectional architecture is designed to capture broader temporal context, comprising two separate temporal convolutional modules, namely, a forward module and a reverse module. The detailed learning and update mechanisms are described as follows:

To specify *X* = {*x*_0_, *x*_1_, …, *x_N_*}, the actual data is first flipped to construct the reversed sequence, as given below.

(14) $$X^{\prime }=\left\{x_{N,}\ldots ,x_{1^{\prime }}x_{0}\right\}$$

Then, the reversed sequences are provided to an inverse TCN for training to extract feature representations.

*c*′ = *TCN*(*x*′) (15)

Here, *c*′ denotes the reverse temporal features. In the final step, an activation function is introduced to carry out a nonlinear transformation and generate the final feature representation.

*h*′ = *σ*(*iVx*′ + *b*) (16)

**Figure 3:**
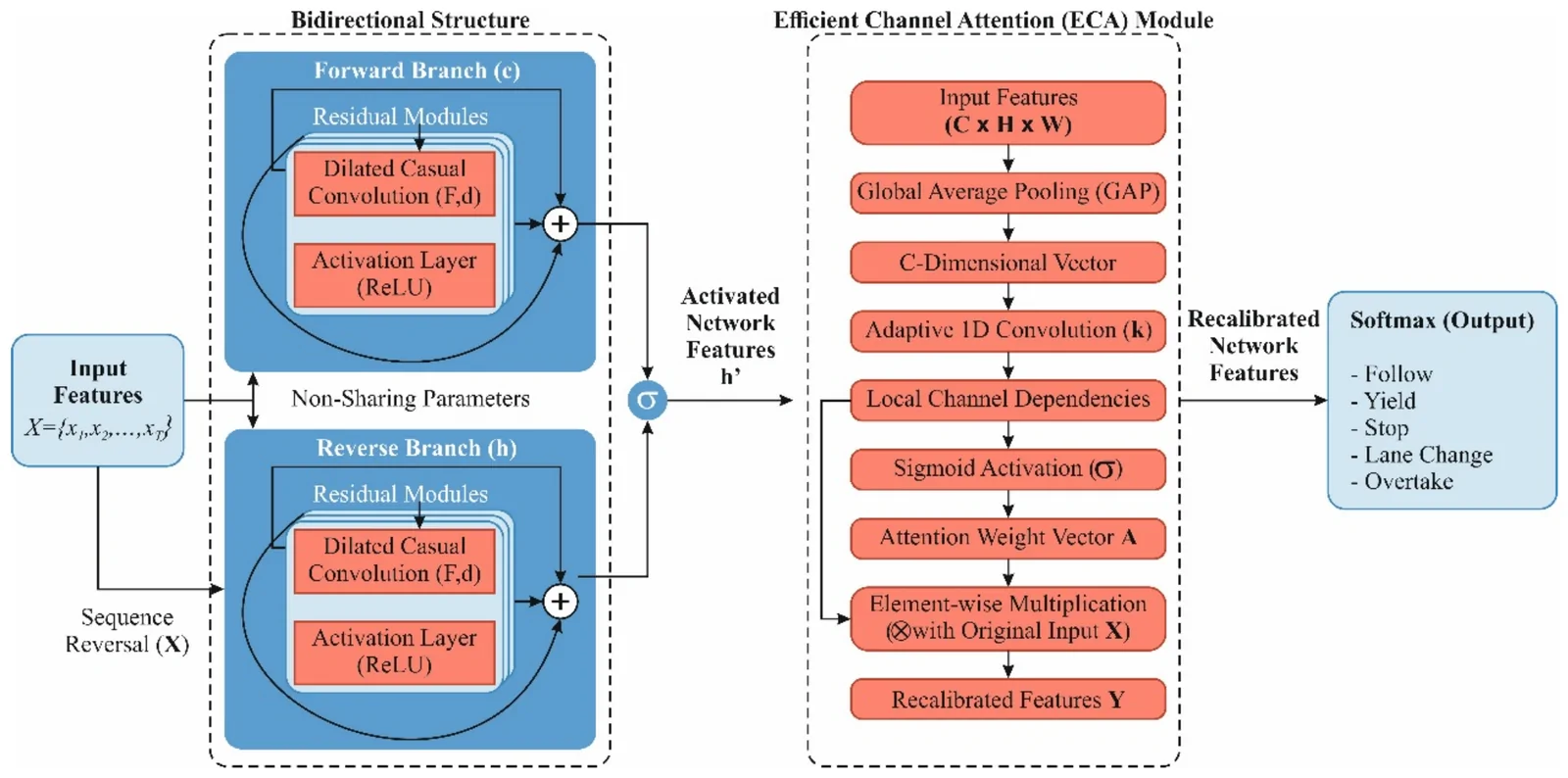
Design of the Bi-TACN model.

In this context, *W* implies the weight matrix, *b* is the bias, *h*′ represents the activated feature, and *σ* is the activation function. Figure 3 depicts the design of the Bi-TACN model.

The Bi-TACN processes each sensor window in both forward and reverse directions, which increases the computational cost compared with a unidirectional TCN. Since the reverse branch uses later observations from the same input window, the model is designed for sequence-based classification rather than strictly causal real-time prediction. For real-time deployment without access to future observations, causal or limited-look-ahead processing would be required.

Efficient Channel Attention: The use of attention mechanisms has greatly improved the effectiveness of models. Many existing approaches emphasize increasingly complex attention modules to enhance performance, but this leads to increased model complexity and computation demands. To achieve lower complexity and improved generalization, a lightweight module named ECA is introduced to reduce computation and prevent overfitting. The ECA module is capable of generating channel-wise weights while learning inter-channel dependencies. Key features in the time series are highlighted with greater weights, while unimportant ones are allotted smaller weights. By means of backpropagation, the model learns to highlight essential temporal patterns and reduce the influence of irrelevant noise. Hence, this framework highlights useful features and increases the network’s sensitivity to important features. Initially, the ECA method uses Global Average Pooling (GAP) for capturing the channel-wise global context. The ECA module was used as a lightweight channel-attention mechanism to capture relationships between different channels without adding many parameters. However, this study does not include a separate comparison of ECA with other attention mechanisms, such as basic channel attention or SE attention. Therefore, the performance of ECA relative to other attention mechanisms has not been established in this study and requires further evaluation.

(17) $$\mathrm{GAP}\left(X\right)=\frac{1}{H\times W}\sum_{i=1j}^{H}\sum_{=1}^{W}X_{\mathrm{cij}}$$

In the formulation, the term *C* denotes the count of channels, *H* signifies the height, and *W* implies the width of the input feature mapping *X*. The term *X_cij_* denotes the element at position (*i*, *j*) in channel *c*. The output is a vector of dimension *C* that summarizes the average response per channel. Later, this method extracts local cross-channel relationships by applying a 1D convolution operation. In the ECA module, the kernel size *k* of the 1D convolution layer is dynamically described. Such flexibility enables ECA to adapt to different channel sizes and more efficiently capture local relationships between channels.

(18) $$k=\left|\frac{\log_{2}C+b}{\gamma }\right|$$

Here, *γ* denotes a parameter controlling the range of local dependency, and *b* is the bias-related parameter. A lower *γ* value results in a larger kernel, allowing a wider range of channel dependencies to be captured. The model follows the default parameter configuration, setting *γ* = 2 and *b* = 1. The output of the 1D convolution layer can be written as:

*ConvlD*(*GAP*(*X*)) = *F_ConvlD_* (*GAP*(*X*)) (19)

Here, *F*_Conv1D_ represents the 1D convolution function with kernel *k*. The outcome is fed into a sigmoid function to generate the attention vector (A).

*A* = *σ*(*Conv lD*(*GAP*(*X*))) (20)

In the final step, channel-attention rescaling is achieved by applying element-wise multiplication between the attention vector *A* and the original input feature map *X*.

*Y_cij_* = *A_c_* ⋅ *X_cij_* (21)

In the above formula, *Y_cij_* represents the feature mapping, where *c* ∈ {1, …, *C*}, *i* ∈ {1, …, *H*}, and *j* ∈ {1, …, *W*}. The contribution of sensor and kinematic features is not uniform, and long temporal histories can introduce noise from unimportant data. To address this, the ECA module applies adaptive weighting across feature channels, highlighting the most relevant driving information. Hence, Bi-TACN gathers extensive temporal information, while the ECA module prioritizes the essential features within that context. This approach strengthens the model’s representation capability and accuracy without greatly increasing computational complexity. The proposed Bi-TACN was evaluated using the complete sensor-kinematic sequences available in the dataset. However, the model was not separately tested under conditions involving incomplete observations, sensor interruptions, or different sequence lengths. These conditions could affect the extraction of temporal features and the resulting classification performance. Further experiments using truncated sequences, missing observations, and varying sequence lengths would help assess the robustness of the model under such conditions.

### 3.4. Model Optimization

The Adamax optimizer is utilized to improve the convergence of the training process and enhance its stability. Adamax is an adaptive moment estimation approach that differs from Adam by extending the parameter update rule using the infinity norm (∞-norm) of previously encountered gradients [25]. Each parameter update in Adamax is rescaled using the infinity norm of historical gradients, which helps improve the stability of the training process. Adamax works effectively with high-dimensional or sparse datasets, where high Lp-norm-based parameter updates with large *p* values could create unstable behavior or increase complexity. However, Adamax provides a simple and stable optimization approach. Unlike Adam, it maintains exponential moving averages of the gradients while using the ∞-norm for scaling instead of the conventional L2-norm. Hence, the algorithm provides stable convergence throughout the training iterations while maintaining computational simplicity and ease of implementation. The first moment estimate of the gradient is updated as:

*mr* ← *β*1.*mr* − 1*m_r_* ← *β*_1_.*m_t_*_−1_(1 − *β*1) (22)

The exponentially weighted infinity norm is updated as:

(23) uτ← max β2.uT−1′gt

The model parameters are then updated as:

(24) $$\theta_{\tau }\leftarrow \theta_{\tau -1}-\left(\frac{\alpha }{1-\beta_{1}^{\tau }}\right).\frac{m_{\tau }}{u_{\tau }}$$

**Figure 4:**
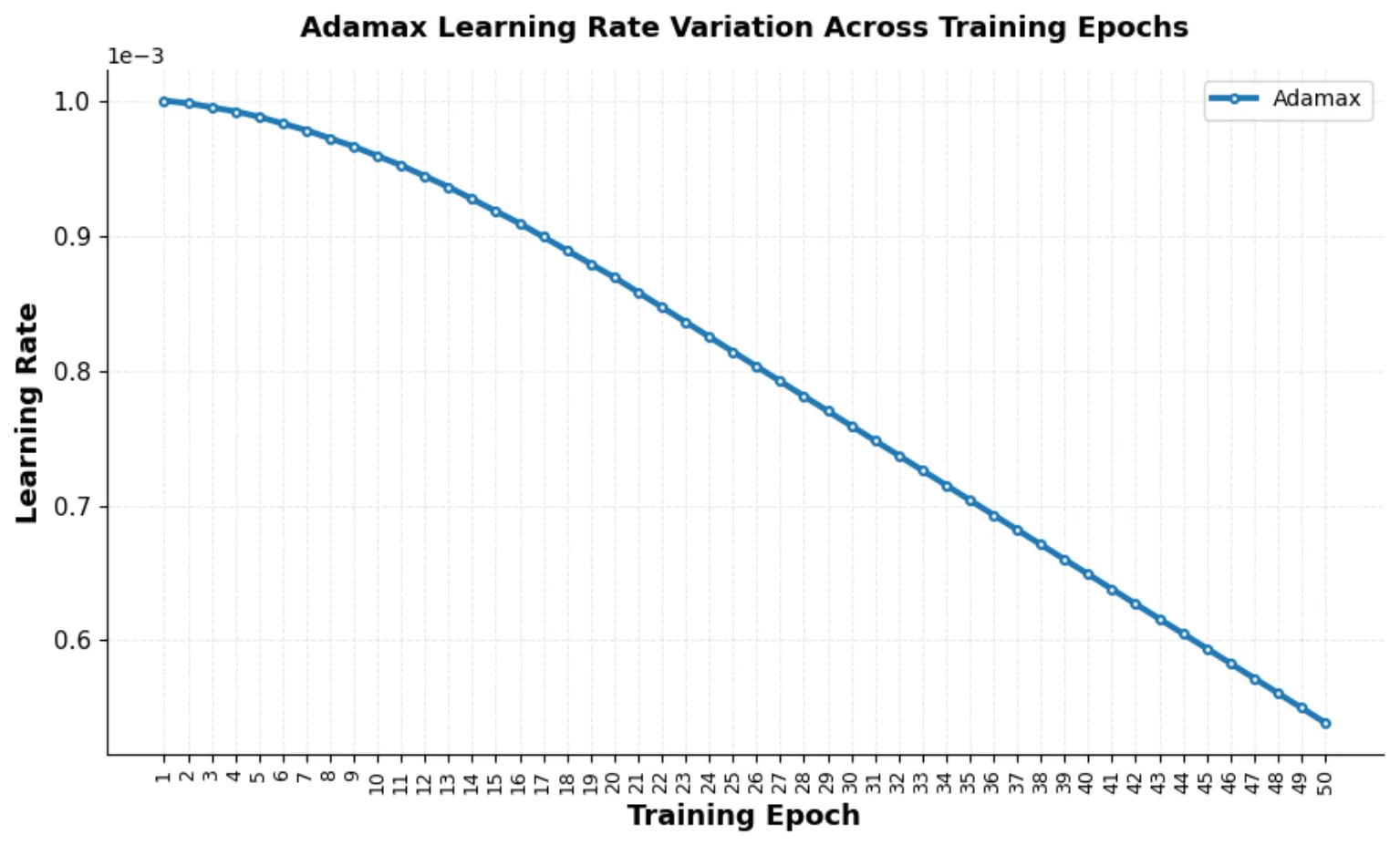
Variation in the Adamax learning rate across 50 training epochs.

Figure 4 shows the gradual decrease in the Adamax learning rate during training. The learning rate decreases from approximately 1.0 × 10^−3^ at the beginning to 0.54 × 10^−3^ by the 50th epoch, denoting a gradual learning rate adjustment for stable model optimization.

The reported performance represents the combined effect of MI-FS, Bi-TACN, and Adamax. However, the specific contribution of Adamax was not evaluated separately. The main model settings are given in Table 2, including 16 selected features (β = γ = 0.5), three TCN layers with 64 filters, a kernel size of 3 and dilation rate of 2, an ECA kernel size of 3, four attention heads, an attention dimension of 64, a dropout rate of 0.3, and a weight decay of 0.0001. Adamax was used with a learning rate of 0.001, a batch size of 32, and 50 training epochs.

Adamax cannot address dataset-related limitations, such as class imbalance and limited data diversity. Therefore, the reported performance is specific to the selected dataset, and further testing on diverse driving datasets is needed to assess the generalizability of the model. The overall workflow of IBTAF-NDCAV is presented in Algorithm 1.
**Algorithm 1.** IBTAF-NDCAV.Input**:** Sensor-kinematic dataset *D* Output: Predicted navigation decision *Y*Input the sensor-kinematic samples along with their corresponding navigation labels.Preprocess the data by performing data cleaning, outlier detection, transformation, and normalization.Initialize the 18 input features and calculate their relevance using mutual information.Assess the marginal and conditional redundancy between the features.Select the 16 most informative features using the improved MI-based feature selection objective.Form the selected feature sequences for temporal modeling.Pass the feature sequences through the forward and reverse TCN branches of Bi-TACN.Apply temporal attention to identify important features and temporal patterns.Combine the bidirectional temporal representations and classify the navigation decision.Optimize the network parameters using the Adamax optimizer.Continue the training process until the specified number of epochs is completed or the model converges.Generate the navigation decision for each input sample.Return the predicted navigation class: Follow, Yield, Stop, Lane Change, or Overtake.

## 4. Experimental Findings and Discussions

The empirical evaluation of the IBTAF-NDCAV method was conducted using the Autonomous Driving Sensor-Kinematics (ADSK) Dataset [26] and the ADAS-EV Dataset [27]. The dataset is a real-time dataset developed to help research and expansion in AV risk prediction and decision-making. This dataset can incorporate simulated sensor data (radar, LiDAR, and camera) with environmental contexts, vehicle kinematics, and navigation-relevant parameters. The dataset is implemented for ML method training and assessment, thus providing balanced traffic conditions, obstacle encounters, and environmental conditions. This dataset is especially valuable for evaluating navigation decision classification under dynamic settings. It is also useful for real-world navigation decision classification, risk prediction and collision systems, and multi-sensor data fusion for AVs. The dataset contains 18 features, of which 16 informative features were selected using the improved MI-based feature selection method. As shown in Table 3, it consists of 5000 samples distributed across five classes: follow**,** yield**,** stop**,** lane change, and overtake**.** To reduce the risk of data leakage from highly correlated consecutive samples, the dataset was split at the driving-sequence level instead of randomly dividing individual samples. Samples from the same driving sequence were kept in the same partition so that temporally adjacent observations did not occur in both the training and test sets. An 80:20 train–test split was then applied to the resulting independent sequence groups. The experiments were performed using an NVIDIA RTX 4090 GPU with 24 GB VRAM, an Intel Core i9 processor, and 32 GB of system RAM on a Windows operating system. The model was implemented in Python 3.11 using the corresponding deep learning framework. The same hardware and software configurations were used across all experiments and throughout the computational evaluation.

The dataset consists of 5000 samples, with an uneven distribution across the classes, particularly for Yield (323) and Overtake (233). This imbalance may affect the classification of the minority classes. In addition, the dataset may not fully represent the variety of conditions encountered in real-world driving. Therefore, an independent external dataset was used for further evaluation, and the corresponding results are presented in the following section.

**Figure 5:**
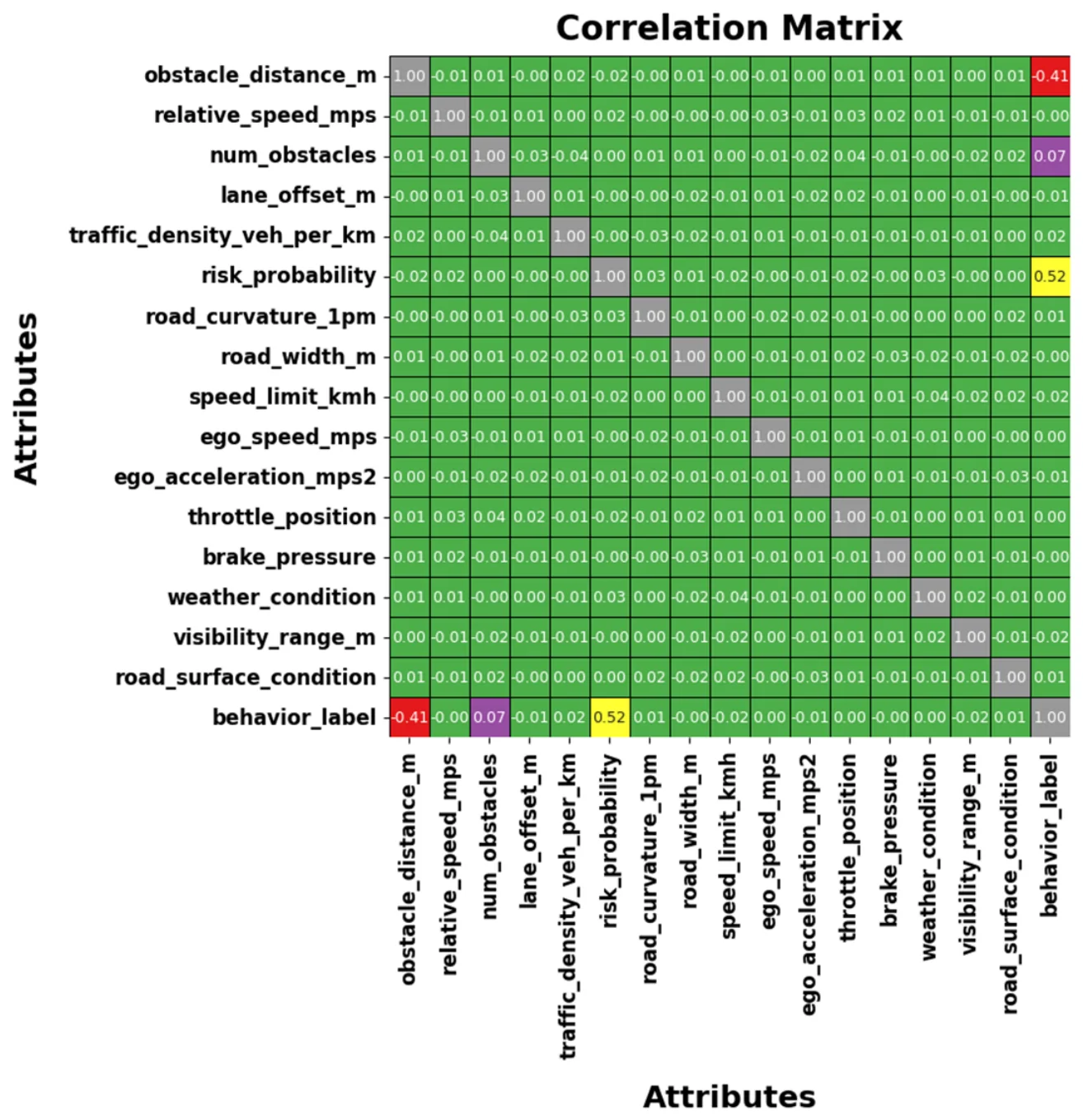
Correlation matrix of the IBTAF-NDCAV approach.

Figure 5 displays the correlation matrix of the IBTAF-NDCAV system, demonstrating the correlations among different driving attributes and the target behavior label. Many attributes show weak relationships with the behavior label, except for risk_probability and obstacle_distance. Such stronger relationships indicate that obstacle distance and risk probability are important factors influencing driving behavior. Also, the matrix exhibits a low level of multicollinearity among the features, thus indicating that the features provide different contributions to the model.

**Figure 6:**
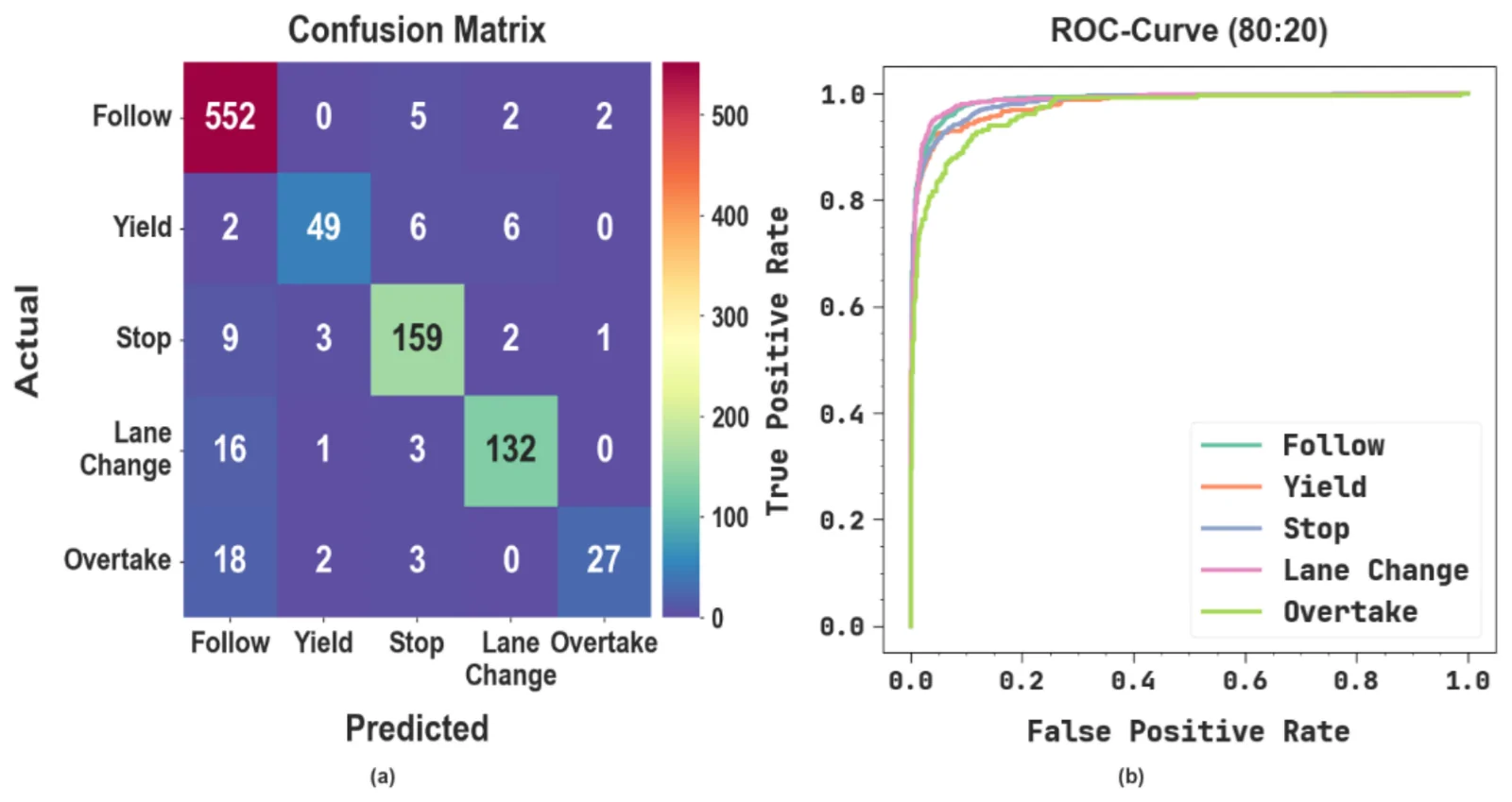
Classifier results of the IBTAF-NDCAV approach: (**a**) confusion matrix and (**b**) ROC-curve with 80:20 split.

Figure 6 displays the classifier outcomes of the IBTAF-NDCAV method. Figure 6a shows the confusion matrix, indicating the classification performance for each class. Similarly, Figure 6b presents the ROC analysis, thereby showing strong classification performance with high ROC values across the different classes.

**Figure 7:**
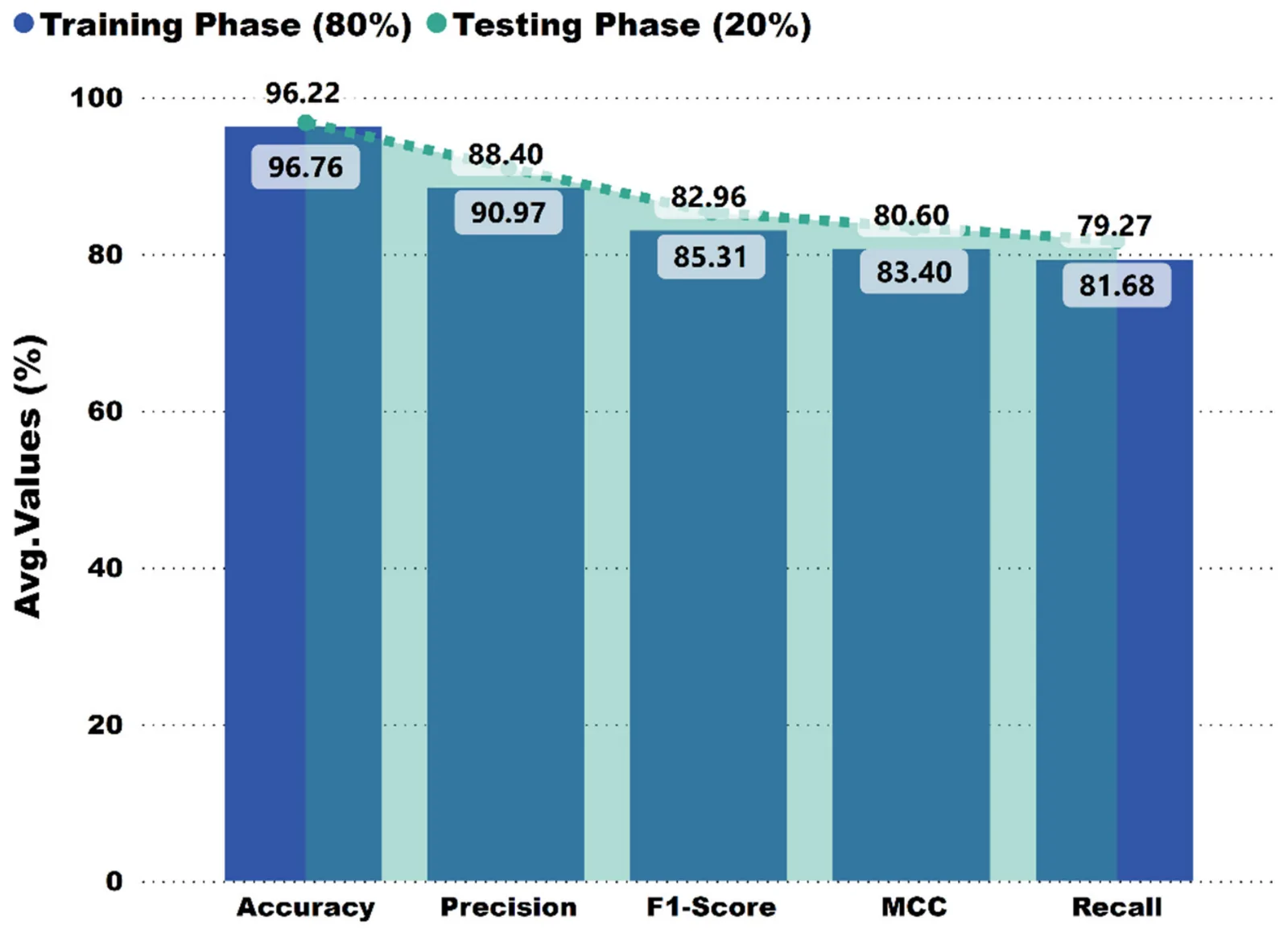
Average value of the IBTAF-NDCAV method with 80:20 split.

Table 4 and Figure 7 represent the AV navigation classification results of the IBTAF-NDCAV approach with an 80:20 training-to-testing data split. The results indicate that the IBTAF-NDCAV method effectively classifies the navigation decision classes. For the 80% training set (TRAP), the IBTAF-NDCAV system accomplishes an average accuracy of 96.22%, precision of 88.40%, recall of 79.27%, F1-score of 82.96%, and MCC of 80.60%, respectively. Meanwhile, for the 20% testing set (TESP), the IBTAF-NDCAV system obtains an average accuracy of 96.76%, precision of 90.97%, recall of 81.68%, F1-score of 85.31%, and MCC of 83.40%, respectively.

**Figure 8:**
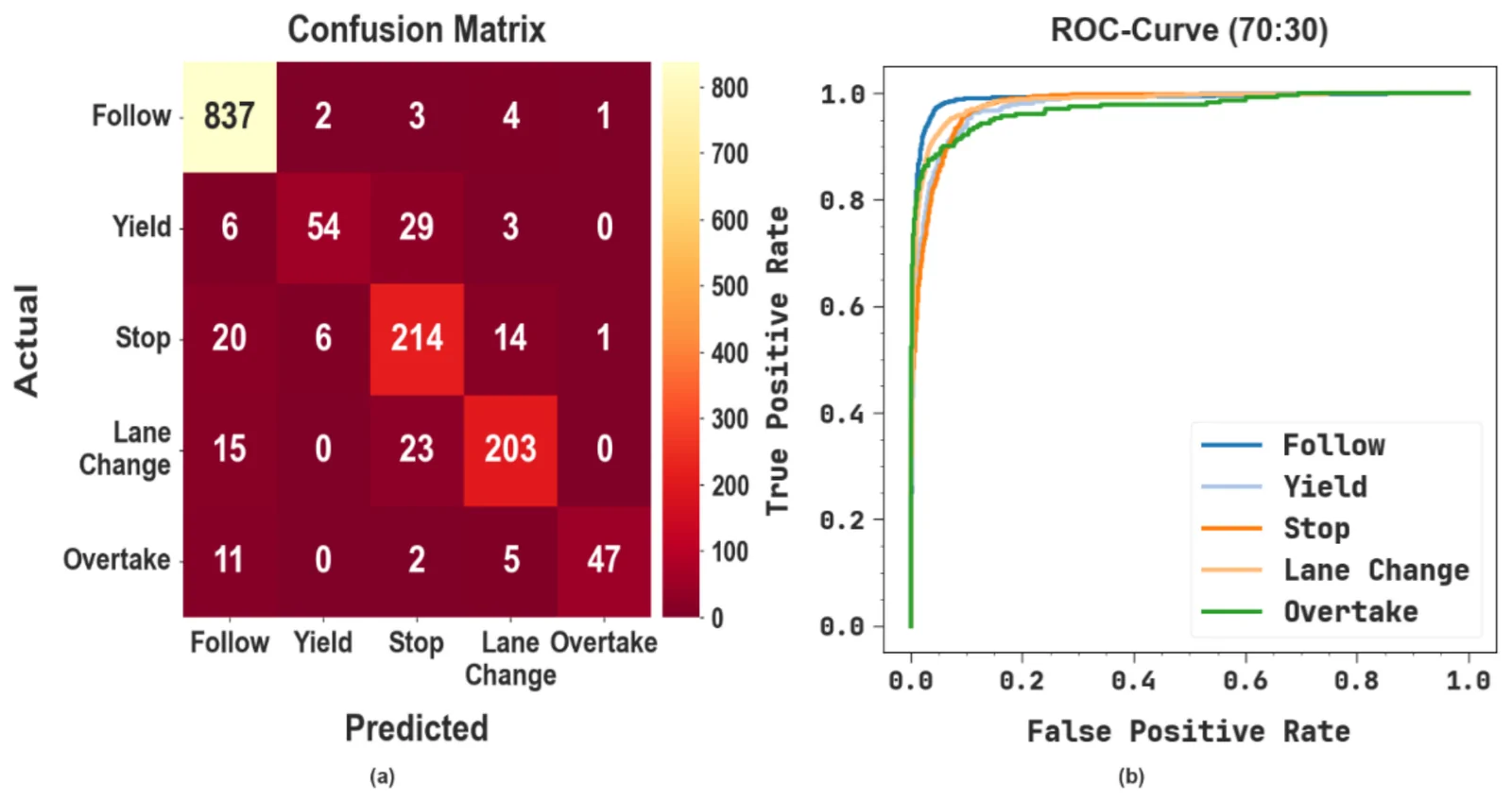
Classifier results of the IBTAF-NDCAV approach: (**a**) confusion matrix and (**b**) ROC-curve with a 70:30 split.

Figure 8 represents the classifier outcomes of the IBTAF-NDCAV method. Figure 8a demonstrates the confusion matrix, which indicates the classification performance for each class. Additionally, Figure 8b shows the ROC analysis with a 70:30 data split, exhibiting strong classification performance with high ROC values across the various classes.

**Figure 9:**
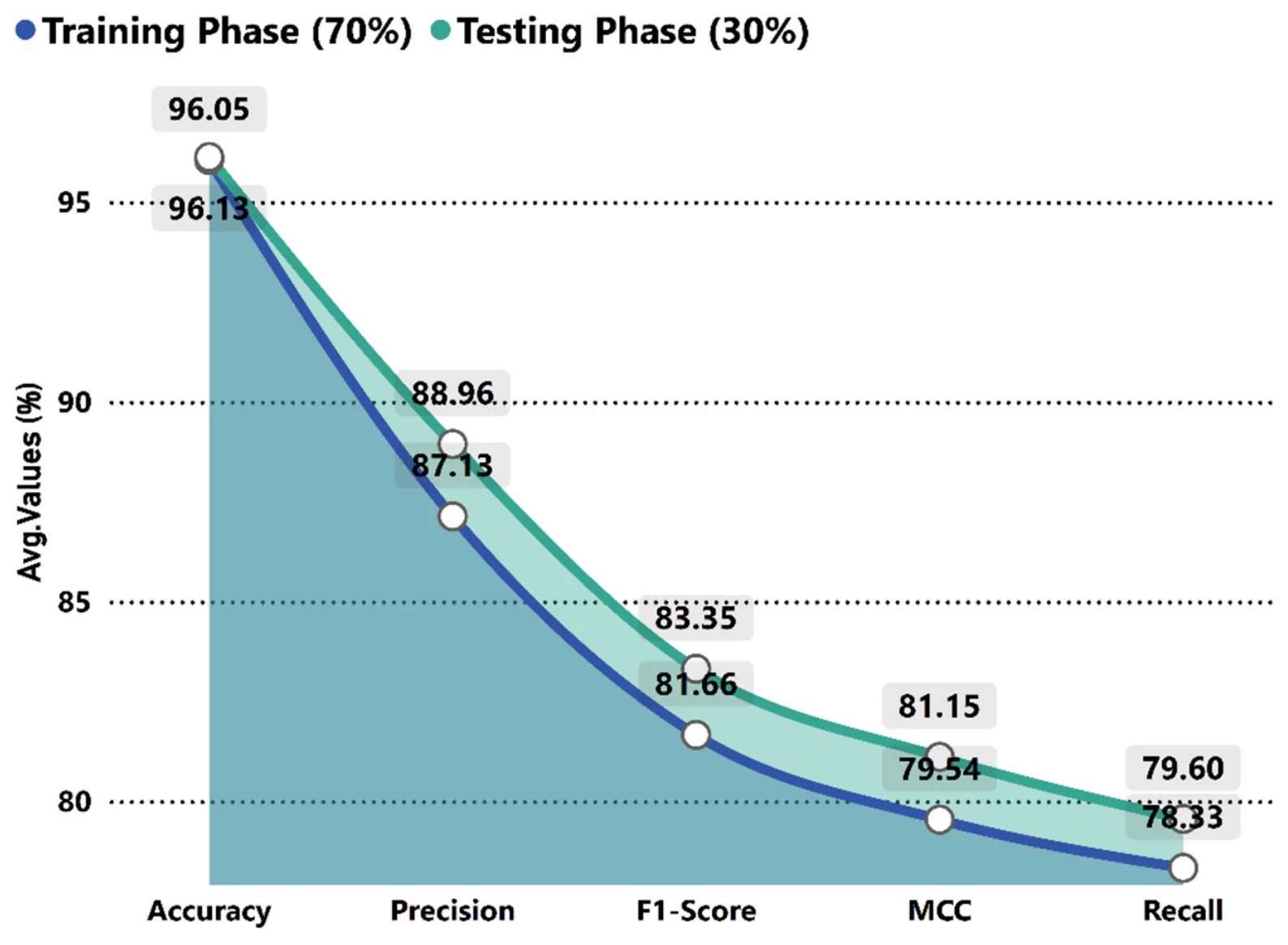
Average value of the IBTAF-NDCAV method with 70:30 split.

Table 5 and Figure 9 display the AV navigation classification outcome of the IBTAF-NDCAV system with a 70:30 training-to-testing data split. The experimental outcome indicates that the IBTAF-NDCAV technique effectively classifies the navigation decision classes. For the 70% training set (TRAP), the IBTAF-NDCAV method obtains an average accuracy of 96.05%, precision of 87.13%, recall of 78.33%, F1-score of 81.66%, and MCC of 79.54%. Similarly, for the 30% testing set (TESP), the IBTAF-NDCAV model attains an average accuracy of 96.13%, precision of 88.96%, recall of 79.60%, F1-score of 83.35%, and MCC of 81.15%. 

The overall recall of 81.68% shows that the classification performance varies across the navigation classes. Overtake, which is the least represented class with 233 samples, has the lowest recall at around 54%, while Yield, with 323 samples, also has a lower recall. This difference may be related to the limited representation of minority classes and the difficulty in learning class-specific patterns. Therefore, recall is considered together with precision, F1-score, and MCC when interpreting the classification results.

The comparative evaluation of the IBTAF-NDCAV system with existing techniques is presented in Table 6 [28,29,30,31,32,33]. The experimental results indicate that the IBTAF-NDCAV technique achieves superior overall performance compared with the existing methods. For a fair comparison, the baseline methods and the proposed IBTAF-NDCAV model were tested using the same dataset, training and testing configuration, and performance metrics. A consistent experimental setup was used throughout the comparison to reduce variations caused by different evaluation conditions. The selected baselines cover different learning approaches for autonomous navigation decision classification, including reinforcement learning methods such as DQN, PPO, and Dueling DQN, as well as deep learning methods such as VGG-16, DQfD-VGG-16, CycleGAN, and ANN. All methods were evaluated under the same conditions to ensure a consistent comparison.

**Figure 10:**
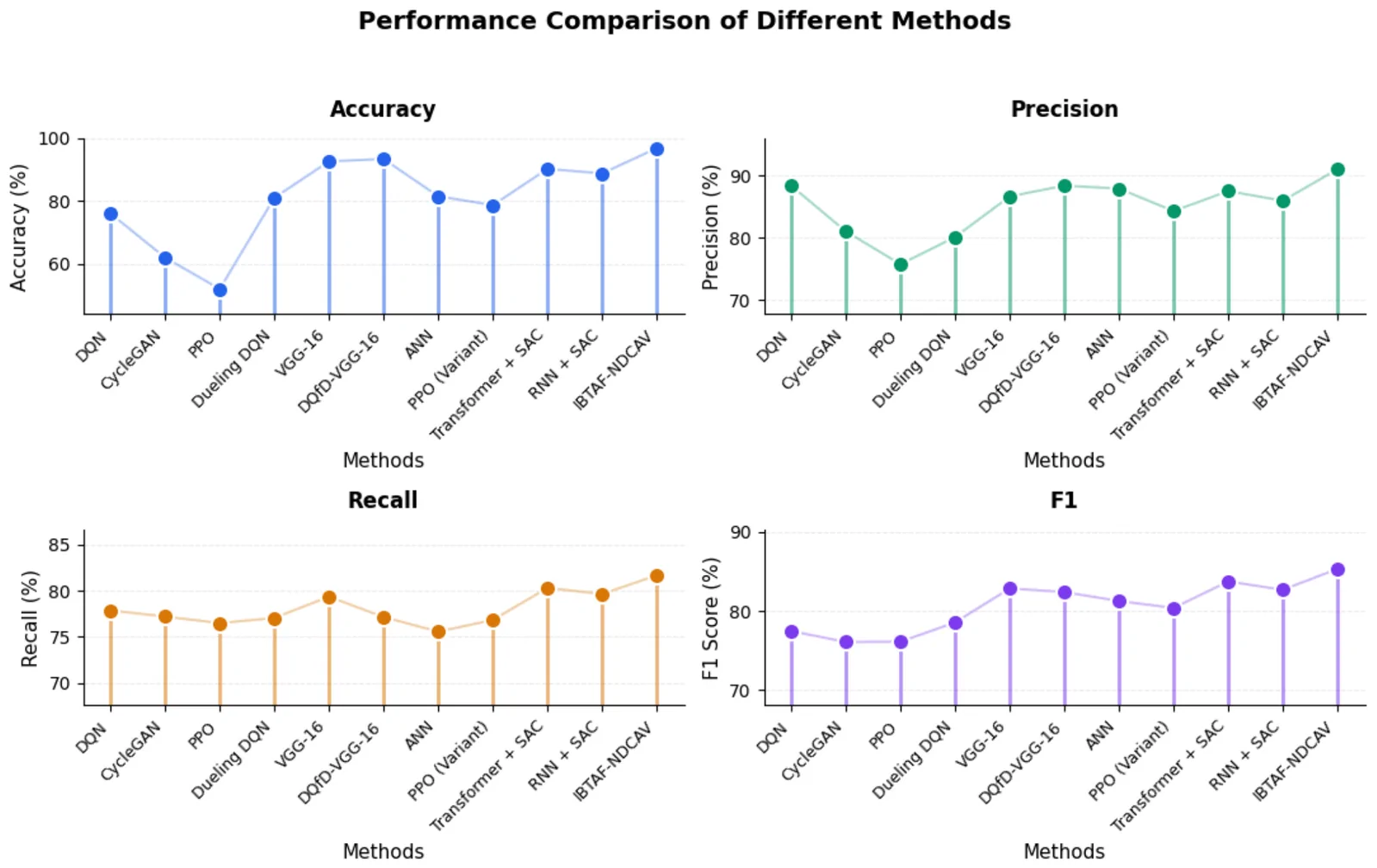
Comparison results of the IBTAF-NDCAV system with metrics.

Figure 10 depicts the comparative study of the IBTAF-NDCAV method with respect to various performance measures. The outcome indicates that the CycleGAN and PPO models achieve the lowest overall performance, while the DQN, PPO, and RNN + SAC methods achieve slightly higher performance. Moreover, the Dueling DQN, VGG-16, DQfD-VGG-16, ANN, and transformer + SAC models achieve reasonable performance. However, the IBTAF-NDCAV approach achieves the highest overall performance, with an accuracy of 96.76%, precision of 90.97%, recall of 81.68%, and F1-score of 85.31%.

**Figure 11:**
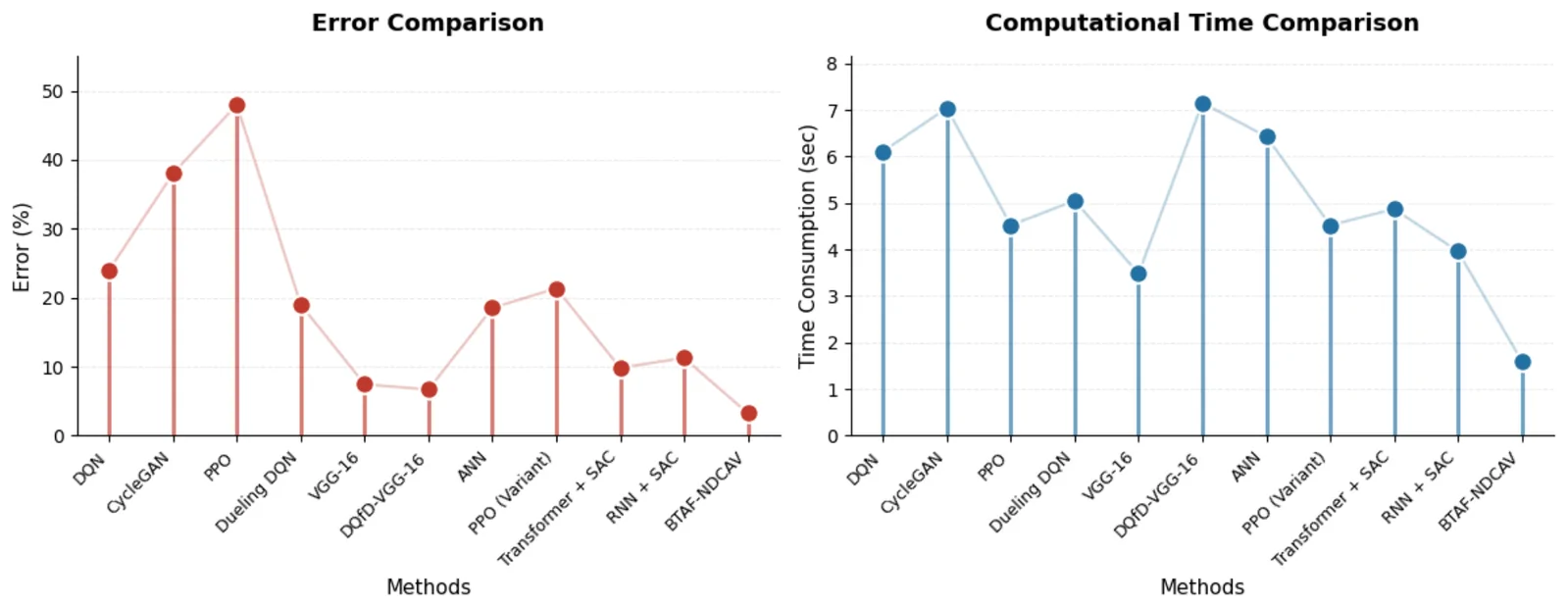
Error and time consumption of the IBTAF-NDCAV approach with other techniques.

Figure 11 displays the error and time consumption of the IBTAF-NDCAV model with existing approaches. Based on the error rate, the proposed IBTAF-NDCAV technique obtains the lowest value, with an error of 3.24%, while the DQN, CycleGAN, PPO, Dueling DQN, VGG-16, DQfD-VGG-16, ANN, and PPO, transformer + SAC, RNN + SAC methodologies attain higher error rates of 24.00%, 38.00%, 48.00%, 19.00%, 7.43%, 6.68%, 18.51%, 21.36%, 9.82%, and 11.27% respectively. Furthermore, the IBTAF-NDCAV method achieves the lowest time consumption of 1.60 s, while the DQN, CycleGAN, PPO, Dueling DQN, VGG-16, DQfD-VGG-16, ANN, PPO, transformer + SAC, RNN + SAC approaches require 6.11 s, 7.04 s, 4.52 s, 5.05 s, 3.48 s, 7.15 s, 6.43 s, 4.52 s, 4.87 s, and 3.96 s, respectively.

The proposed IBTAF-NDCAV model was further evaluated on the ADAS-EV dataset to examine its performance on an additional dataset. As reported in Table 7, the model achieves 96.42% accuracy, 96.31% precision, 96.18% recall, and a 96.24% F1-score. The comparison methods, including TabM, TabPFN-v2, FT-transformer, TabR, SAINT, AutoInt, and DANets, show lower values across the evaluated metrics. The results indicate that IBTAF-NDCAV provides consistent classification performance when tested on the additional ADAS-EV dataset.

Table 8 shows the sensitivity analysis of the β and γ parameters and their effects on accuracy, precision, recall, and F1-score. The best performance is obtained with β = 0.6 and γ = 0.5, achieving an accuracy of 96.21% and an F1-score of 84.92%. The results suggest that moderate values of these parameters provide better classification performance, whereas increasing β or γ further results in a slight decrease in performance.

An ablation study was conducted to examine the contribution of the main components of IBTAF-NDCAV. The experiments progressively included Improved MI-FS, ECA, and temporal attention, along with a comparison between Adam and Adamax. TCN and Bi-TCN configurations without feature selection were also considered. As shown in Table 9, the complete Bi-TCN + Improved MI-FS + ECA + temporal attention configuration performed better with Adamax than with Adam. The full IBTAF-NDCAV model achieved 96.76% accuracy, 90.97% precision, 81.68% recall, and 85.31% F1-score, showing the combined effect of feature selection, attention mechanisms, and optimization. 

Table 10 exhibits the computational efficiency of the IBTAF-NDCAV approach compared to other techniques, including FLOPs, GPU usage, and inference time. Based on inference time, the IBTAF-NDCAV system achieves the lowest inference time of 0.25 ms, with 3.94M FLOPs and 815M GPU usage, whereas the BiLSTM, Transformer Encoder, EGCN, YOLOv7X, YOLOv7X + MPDIoU, and YOLOv7X + CBAM approaches require higher inference times of 2.55 ms, 8.29 ms, 4.87 ms, 2.67 ms, 6.27 ms, and 7.93 ms, respectively.

FLOPs, GPU utilization, memory usage, and inference time were measured using the specified GPU, while power consumption was not evaluated. The reported inference time of 0.25 ms is specific to the hardware used in the experiments and should not be considered as validated in-vehicle latency. Further evaluation on automotive-grade hardware would be required to assess the actual deployment performance. Table 11 presents the results of the Wilcoxon signed-rank test, which was used to examine whether the differences in F1-score between IBTAF-NDCAV and the comparison models were statistically significant. The *p*-value obtained for all comparisons was 0.00195, which is below the commonly used significance level of 0.05. Thus, the observed differences are statistically significant, indicating that the F1-score of IBTAF-NDCAV differs significantly from those of the evaluated baseline and ablation models.

Table 12 illustrates the k-fold cross-validation results of the proposed IBTAF-NDCAV method. The model obtained accuracy values ranging from 96.52% to 96.91%, with the highest accuracy of 96.91% in Fold 4. A better precision of 91.28%, a recall of 82.02%, and an F1-score of 85.66% were achieved in Fold 4. Although there were minor variations across the folds, the continuously better evaluation metrics demonstrate the stability and strong generalization capability of the proposed algorithm. Furthermore, the mean values of 96.71% accuracy, 90.85% precision, 81.63% recall, and 85.25% F1-score specify the reliable outcome of the proposed IBTAF-NDCAV model over all folds. The corresponding standard deviations (Std. Dev.) of 0.12, 0.27, 0.25, and 0.25, respectively, depict few performance differences, confirming the model’s stability and robustness.

The improved performance of the proposed IBTAF-NDCAV model is accomplished by employing the synergistic integration of FS method, classification, and optimization approaches. For feature selection, an improved MI-based technique is utilized to remove less informative features while reducing redundancy, thereby ensuring that important navigation parameters such as trajectory data, obstacle distance, and vehicle speed are retained. For classification, the bidirectional temporal attention convolutional network efficiently obtains the forward and backward temporal dependencies, thereby allowing the model to learn intricate patterns in road situations and dynamic traffic. 

With a combination of the improved method for FS (improved MI-based method) to remove less discriminative features and eliminate redundancy, this is a promising way to ensure that navigation-relevant information, such as trajectory information, obstacle distance, risk probability, and vehicle speed, is retained. Using a bidirectional temporal attention convolutional network for classification allows the extraction of both forward and backward dependencies for temporal modeling, allowing the navigation decision and driving model to learn complex driving contexts. The Adamax optimizer is employed to improve convergence stability and provide adaptive learning-rate updates during neural network training. It can be concluded that, with this joint usage of improved feature selection, bidirectional temporal feature learning, ECA-based channel attention, and Adamax-based optimization, the framework can effectively model local and global temporal patterns and assist in reliable navigation decision classification. Although IBTAF-NDCAV achieves high overall performance, the recall is lower for the minority classes, particularly Overtake (54%) and Yield. This may be related to their smaller number of samples, as Follow has 2812 samples compared with 323 for Yield and 233 for Overtake. In addition, the model was not separately evaluated under adverse weather or unusual traffic conditions, so its robustness under such conditions remains to be examined.

## 5. Conclusions

This article presented the IBTAF-NDCAV technique for developing an autonomous driving navigation decision classification system using sensor information. The IBTAF-NDCAV method follows a sequence of steps in which the collected data are cleaned, checked for outliers, transformed, and normalized before further processing. After preprocessing, an improved MI-based FS method is used to select the most relevant features and reduce redundant information. A Bi-TACN is then used to learn temporal dependencies from the sensor data and classify the appropriate navigation decisions. The Adamax optimizer is also used to improve the training process and convergence. The performance of the IBTAF-NDCAV method is evaluated using the benchmark Autonomous Driving Sensor-Kinematics dataset, where it achieves an accuracy of 96.76% and performs better than the existing methods. However, the proposed system still has some limitations. The benchmark dataset is relatively small, which may affect the generalizability of the system to different driving conditions, environments, and traffic patterns. Although an external dataset was included for additional evaluation, further testing on larger and more diverse datasets is needed to assess the model’s generalizability. In addition, although the Bidirectional TA framework can capture complex temporal dependencies, its computational requirements may be challenging for in-vehicle systems with limited resources. The current framework does not provide explicit explanations for individual navigation decisions. In addition, its performance in real-world vehicle deployment and on-road conditions has not yet been fully evaluated. Future work can therefore focus on testing the model with larger and more diverse datasets, developing lightweight and energy-efficient models, and incorporating XAI methods to improve the robustness and interpretability of autonomous navigation decisions in challenging driving environments. Future work will focus on real-world deployment and testing the model under a wider range of driving conditions.

## Figures and Tables

**Table 1:** Related work comparison in autonomous vehicles.

References	Years	Aims	Techniques	Datasets	Results
Almadhor et al. [13]	2026	Improve perception in autonomous driving using multi-modal data.	ResNet-CNN, transformer, and Grad-CAM.	nuScenes dataset	mAP of 72.8.
Huang et al. [14]	2020	Enable end-wise autonomous driving with scene understanding.	DNN model.	CoRL2017 and NoCrash datasets	Enhanced scene understanding and driving efficiency.
Sethuraman et al. [15]	2025	Improve vehicle performance using a cloud-based optimizer.	SVM, RF, ABWO, and AdaBoost algorithms.	Context 2017 dataset	Accuracy of 97.82%.
Gondhalekar et al. [16]	2025	Explore AI uses in autonomous vehicle systems.	CNN-ViT and RL techniques.	KITTI, Waymo, and nuScenes	Accuracy of 97.3%.
Mishra et al. [17]	2025	Improve robust navigation method using attention mechanisms.	DNN with attention and FCNN model.	nuScenes and LeddarTech PixSet Datasets	Accuracy of 95.3%.
Sugumar et al. [18]	2026	Enhance object detection and navigation using IoT integration.	TS-CDOA, BiRNN, and DDQN methods	Sensor-based IoT datasets	Accuracy of 98.95%.
Aloqaily et al. [19]	2025	Detect intrusion attacks in autonomous vehicles.	ML approaches.	Network traffic datasets	Accuracy of 99.9%.
Ge et al. [20]	2024	Enhance autonomous driving decisions in partially observable environments.	SAC, decision transformer, VAE.	Urban driving environment	Obtained better policies than baseline techniques.
DRHu [21]	2023	Incorporate perception, prediction, and planning for autonomous driving.	Integrated multi-task framework, transformer, query-driven task interaction.	nuScenes	Better autonomous driving planning methods across multiple tasks.

**Table 2:** Parameter settings of the proposed IBTAF-NDCAV model.

Component	Parameter	Setting
Feature Selection	Feature selection method	Improved MI-FS
	Selected feature dimension	16
	β	0.5
	γ	0.5
Temporal Feature Extraction	Architecture	Bi-TCN
	Number of TCN layers	3
	Filters per layer	64
	Kernel size	3
	Dilation rate	2
	Activation function	ReLU
Channel Refinement	Attention module	ECA
	ECA kernel size	3
Temporal Attention	Attention mechanism	Multi-head temporal attention
	Number of attention heads	4
	Attention dimension	64
Regularization	Dropout rate	0.3
	Weight decay	0.0001
Classification	Classifier	Fully connected layer
	Output function	Softmax
	Loss function	Cross-Entropy Loss
Optimization	Optimizer	Adamax
	Initial learning rate	0.001
Training	Batch size	32
	Maximum epochs	50

**Table 3:** Details of ADSK dataset.

Class Labels	No. of Samples
Follow	2812
Yield	323
Stop	893
Lane Change	739
Overtake	233
Total Samples	**5000**

**Table 4:** AV navigation classifier outcome of the IBTAF-NDCAV method with 80:20 split.

Classes	*Accur_y_*	Preci_n_	Recal_l_	F1_score_	MCC
TRAP (80%)					
Follow	94.13	91.90	98.22	94.95	88.22
Yield	97.65	89.52	72.31	80.00	79.27
Stop	95.40	89.28	84.56	86.86	84.12
Lane Change	96.57	88.40	88.25	88.32	86.31
Overtake	97.35	82.91	53.01	64.67	65.08
Average	96.22	88.40	79.27	82.96	80.60
TESP (20%)					
Follow	94.60	92.46	98.40	95.34	89.18
Yield	98.00	89.09	77.78	83.05	82.21
Stop	96.80	90.34	91.38	90.86	88.92
Lane Change	97.00	92.96	86.84	89.80	88.11
Overtake	97.40	90.00	54.00	67.50	68.59
Average	96.76	90.97	81.68	85.31	83.40

**Table 5:** AV navigation classifier outcome of the IBTAF-NDCAV method with 70:30 split.

Classes	*Accur_y_*	Preci_n_	Recal_l_	F1_score_	MCC
TRAP (70%)					
Follow	96.09	94.80	98.42	96.58	92.10
Yield	96.34	84.56	54.55	66.32	66.21
Stop	93.89	81.09	86.68	83.79	80.09
Lane Change	95.91	85.01	86.55	85.77	83.39
Overtake	98.00	90.16	65.48	75.86	75.89
Average	96.05	87.13	78.33	81.66	79.54
TESP (30%)					
Follow	95.87	94.15	98.82	96.43	91.68
Yield	96.93	87.10	58.70	70.13	70.07
Stop	93.47	78.97	83.92	81.37	77.46
Lane Change	95.73	88.65	84.23	86.38	83.89
Overtake	98.67	95.92	72.31	82.46	82.66
Average	96.13	88.96	79.60	83.35	81.15

**Table 6:** Comparison results of the IBTAF-NDCAV approach with existing systems on ADSK dataset.

Methods	*Accur_y_*	Error (%)	Preci_n_	Recal_l_	F1_score_	Time Consuming (s)
DQN	76.00	24.00	88.32	77.86	77.46	6.11
CycleGAN	62.00	38.00	81.01	77.20	76.09	7.04
PPO	52.00	48.00	75.75	76.49	83.87	4.52
Dueling DQN	81.00	19.00	80.11	77.05	77.69	5.05
VGG-16	92.57	7.43	86.61	79.36	78.14	3.48
DQfD-VGG-16	93.32	6.68	88.34	77.15	82.14	7.15
ANN	81.49	18.51	87.86	75.56	76.82	6.43
PPO	78.64	21.36	84.27	76.83	80.38	4.52
Transformer + SAC	90.18	9.82	87.46	80.31	83.72	4.87
RNN + SAC	88.73	11.27	85.92	79.64	82.66	3.96
IBTAF-NDCAV	96.76	3.24	90.97	81.68	85.31	1.60

**Table 7:** Cross-dataset performance comparison of IBTAF-NDCAV with existing tabular deep learning methods on the ADAS-EV dataset.

Model	*Accur_y_*	Preci_n_	Recal_l_	F1_score_
TabM	91.84	91.62	91.37	91.48
TabPFN-v2	93.21	93.08	92.86	92.97
FT-transformer	89.76	89.43	89.18	89.30
TabR	92.68	92.51	92.34	92.42
SAINT	88.94	88.71	88.53	88.62
AutoInt	87.63	87.42	87.16	87.29
DANets	90.57	90.31	90.08	90.19
IBTAF-NDCAV	96.42	96.31	96.18	96.24

**Table 8:** Sensitivity analysis of β and γ parameters on the classification performance of the proposed model on ADSK dataset.

β	γ	Accuracy	Precision	Recall	F1
0	0.5	94.82	87.54	77.26	81.94
0.2	0.5	95.31	88.46	78.64	83.01
0.4	0.5	95.84	89.32	79.83	84.16
0.6	0.5	96.21	90.08	80.72	84.92
0.8	0.5	95.73	89.61	79.56	84.07
1	0.5	95.18	88.73	78.81	83.25
0.5	0	95.27	88.41	78.52	82.94
0.5	0.2	95.66	89.17	79.41	83.79
0.5	0.4	96.03	89.82	80.26	84.58
0.5	0.6	96.18	90.01	80.61	84.83
0.5	0.8	95.79	89.46	79.74	84.21
0.5	1	95.34	88.84	78.93	83.43

**Table 9:** Ablation study analysis of the proposed method with ADSK dataset.

Methods	*Accur_y_*	Preci_n_	Recal_l_	F1_score_
TCN (without feature selection and parameter tuning)	90.00	81.52	69.81	74.08
Bi-TCN (without feature selection and parameter tuning)	90.65	82.11	70.03	75.44
TCN + Improved MI-FS	91.84	83.27	70.96	76.58
Bi-TCN + Improved MI-FS	93.21	85.46	73.84	79.12
Bi-TCN + Improved MI-FS + ECA	94.38	87.21	76.25	81.34
Bi-TCN + Improved MI-FS + ECA + Temporal Attention	95.17	88.63	78.14	83.02
IBTAF-NDCAV (Bi-TCN + Improved MI-FS + ECA + Temporal Attention + Adam)	96.08	90.14	80.47	84.72
IBTAF-NDCAV (Bi-TCN + Improved MI-FS + ECA + Temporal Attention + Adamax)	96.76	90.97	81.68	85.31

**Table 10:** Computational efficiency of the IBTAF-NDCAV method compared with existing approaches on ADSK dataset.

Models	FLOPs (M)	GPU (M)	Inference Time (ms)
BilSTM	6.97	4571	2.55
Transformer Encoder	16.73	4550	8.29
EGCN	428.97	2616	4.87
YOLOv7X	188	2125	2.67
YOLOv7X + MPDIoU	188	3471	6.27
YOLOv7x + CBAM	195.1	2446	7.93
IBTAF-NDCAV	3.94	815	0.25

**Table 11:** Wilcoxon signed-rank test results comparing the proposed IBTAF-NDCAV method with ADSK dataset.

Comparison	Metric	Wilcoxon Statistic (W)	*p*-Value	Significance
IBTAF-NDCAV vs. TCN	F1	0	0.00195	Significant
IBTAF-NDCAV vs. TCN + Improved MI-FS	F1	0	0.00195	Significant
IBTAF-NDCAV vs. Bi-TCN + Improved MI-FS	F1	0	0.00195	Significant
IBTAF-NDCAV vs. Bi-TCN + Improved MI-FS + ECA	F1	0	0.00195	Significant
IBTAF-NDCAV vs. Bi-TCN + Improved MI-FS + ECA + Temporal Attention	F1	0	0.00195	Significant

**Table 12:** Cross-validation analysis of the proposed method on ADSK dataset.

Fold	Accuracy	Precision	Recall	F1
1	96.52	90.43	81.21	84.86
2	96.81	91.12	81.83	85.47
3	96.63	90.71	81.46	85.13
4	96.91	91.28	82.02	85.66
5	96.74	90.89	81.71	85.29
6	96.58	90.52	81.32	84.97
7	96.79	91.03	81.88	85.42
8	96.69	90.76	81.59	85.21
9	96.83	91.08	81.77	85.38
10	96.63	90.68	81.49	85.09
Mean	96.71	90.85	81.63	85.25
Std. Dev.	0.12	0.27	0.25	0.25

## Data Availability

The data that support the findings of this study are openly available in the Kaggle repository at https://www.kaggle.com/datasets/ziya07/entrepreneurial-ability-assessment-dataset/data (accessed on 28 September 2026) and https://www.kaggle.com/datasets/ziya07/adas-ev-dataset (accessed on 21 July 2026), reference numbers [25,26].
